# BELIEFS REGARDING GERIATRICS PRIMARY CARE TOPICS AMONG MEDICAL STUDENTS AND INTERNAL MEDICINE RESIDENTS

**DOI:** 10.1093/geroni/igz038.1092

**Published:** 2019-11-08

**Authors:** Dylan J Jester, Kathryn Hyer, Ross Andel

**Affiliations:** 1 School of Aging Studies, University of South Florida, Tampa, Florida, United States; 2 University of South Florida, Tampa, Florida, United States

## Abstract

Our study evaluated and contrasted responses to 25 content areas essential to the primary care of older adults by medical students and residents, and identified attitudes toward aging amongst students and residents. One hundred and thirty-six medical students and 61 Internal Medicine residents completed a survey including the 25-item Geriatrics Clinician-Educator Survey and 18-item Images of Aging Scale. Students and residents rated importance and knowledge for content areas from 1 (low) to 10 (high). Gap scores reflecting the difference in ratings between importance and knowledge were calculated. The Images of Aging scale ranges between 0 (furthest from what you think) and 6 (closest to what you think). Results indicated that students and residents reflected similar beliefs about the importance of content areas, but students provided lower ratings in knowledge. Students revealed larger gap scores in areas that reflected general primary care (e.g., assess chronic conditions, medications), whereas residents revealed larger gap scores in areas that reflected specialists’ expertise (e.g., driving risk, cognition, psychiatric symptoms). Attitudes toward older adults did not differ appreciably between students and residents. In sum, primary care topics applicable for any age demographic were rated as most important by first-year medical students and Internal Medicine residents. Topics relevant to older populations – particularly those requiring specialists’ knowledge of or requiring sensitive discussion with older adults – were rated as less important and were less well mastered.

